# Patterns of health care utilization of gout patients in Hawai‘i-high rates of emergency department utilization as compared to rheumatoid arthritis

**DOI:** 10.1371/journal.pone.0220978

**Published:** 2019-08-15

**Authors:** Victoria P. Mak, Andrea M. Siu, So Yung Choi, Hyeong Jun Ahn, Sian Yik Lim

**Affiliations:** 1 Saint Louis University, St. Louis, Missouri, United States of America; 2 Hawai‘i Pacific Health Research Institute, Honolulu, HI, United States of America; 3 Department of Complementary and Integrative Medicine, University of Hawai‘i, Honolulu, HI, United Sttaes of America; 4 Straub Clinic, Hawai‘i Pacific Health, Honolulu, HI, United States of America; Medical University Graz, AUSTRIA

## Abstract

Recent and comprehensive research of gout in the Pacific region and Hawai‘i is significantly lacking. This study was conducted to improve the understanding of the healthcare utilization of gout patients within a single health care system in Hawai‘i. The objective was to examine gout inpatient, outpatient and emergency department care within a single health care system in Hawai‘i. This study was a retrospective chart review of patients, ≥ 18 years admitted to three Hawai‘i Pacific Health facilities for a primary diagnosis of gout or rheumatoid arthritis (RA) from 2011 to 2017. Population data for the State of Hawai‘i was used to calculate visit rates per 1,000 Hawai‘i adults. Trend analysis was performed to compare changes over time. We studied gout health care utilization concurrently with RA to provide an internal comparison group for the healthcare utilization patterns of interest. Gout patients were primarily managed in the outpatient setting with high rates of emergency department visits. In contrast, RA patients were primarily managed in the outpatient setting, with low rates of emergency department visits. Both gout and RA patients had low rates of inpatient admissions. The cost of gout emergency department visits was approximately 3.4 times higher than gout outpatient visits. The rates for gout emergency department visits, outpatient visits, inpatients visits, and RA outpatient visits in 2017 were trending downward and significantly changed from 2011 (p <0.05). The rates for RA emergency department visits and inpatient visits were not significantly changed from 2011–2017. Gout care in Hawai‘i remains suboptimal with higher rates of emergency department visits, as compared to RA. Because emergency department visits are associated with higher cost, efforts should be made to reduce these emergency department visits to improve the quality of care.

## Introduction

Gout is one of the most common inflammatory arthritides [1]. The incidence of gout is increasing [2], and recent studies have described an increasing burden of gout in the United States due to hospitalizations, emergency department visits and cost associated with suboptimal gout care [1, 3, 4]. Prior studies have focused solely on emergency department visits [3], or hospital admissions [1], however, there is scarce information about patterns of gout patient health care utilization within a single health care system.

Hawaiʻi has a distinct racial composition (compared to the contiguous United States, the racial composition in the state of Hawaiʻi includes a higher proportion of Asians, Pacific Islanders, and multi-racial persons), and differs geographically and culturally from the rest of the United States [5]. Major ethnocultural groups including Native Hawaiians, Filipinos, and Japanese are thought to have increased uric acid levels and therefore increased risk for gout [6]. Despite the strong need for research into gout in Hawai‘i, recent and comprehensive research of gout in the Pacific region, and Hawai‘i is significantly lacking [6]. The lack of data specific to the region present significant challenges in assessing the disease burden of gout and formulating public health policies and efforts to improve care [6].

To address these key knowledge gaps, we investigated gout inpatient, outpatient and emergency department care within a single health care system in Hawai‘i between 2011–2017 as compared to rheumatoid arthritis (RA). We studied gout patient health care utilization concurrently with RA patients to provide an internal comparison group for the healthcare utilization patterns of interest.

## Methods

### Research setting & design

We conducted a retrospective chart review of gout and RA patients at Hawai‘i Pacific Health, one of the largest healthcare providers in Hawai‘i. Hawaiʻi Pacific Health is a not-for-profit healthcare network of hospitals, clinics, physicians, and other care providers that covers the state of Hawai‘i [7]. Through review of medical records, the authors had access to identifying information. The study was reviewed and determined to be exempt from Institutional Review Board approval by the Hawai‘i Pacific Health Research Institute.

### Study participants and analysis variables

We included patients seen in the Hawaiʻi Pacific Health healthcare system of at least 18 years of age who were seen in the outpatient setting, emergency department, or had an hospital admission at various facilities of Hawaiʻi Pacific Health (Straub Medical Center, Pali Momi Medical Center, and Wilcox Memorial Hospital) with a primary discharge diagnosis of gout (International Classification of Diseases, 9^th^ Revision, Clinical Modification [ICD-9-CM] codes: 274.0–274.9) or RA (ICD-9-CM codes: 714.0, 714.2, 714.30–714.33) from January 1^st^ 2011 to December 31^st^ 2017. We excluded patients with less than one year of follow-up data. Data extracted from the electronic medical record system included age, gender, race, treatment facility, and insurance type of the patient at the time of admission.

Rheumatoid arthritis was used as an internal comparison group because both rheumatoid arthritis and gout are one of the most common inflammatory arthritides [1]. In recent years, improvement of rheumatoid arthritis care, has led to reported decreases in patient hospitalizations and emergency department utilization [1, 8]. In contrast, gout care remains suboptimal, with increasing prevalence and incidence over the past several decades [1]. We investigated both gout and rheumatoid arthritis, because we hypothesized contrasting health care utilization patterns for both conditions, with each condition serving as an internal comparison group.

### Statistical analyses

We examined and stratified gout and rheumatoid arthritis patients by subgroups, including age (18–44, 45–64, 65–84, >85 years), sex, race, and insurance status for the study period 2011–2017. Changes during the study period were assessed using percentages of categories within each variable by each year. We calculated annual population rates of outpatient visits, inpatient admissions, and emergency department visits (per 1000 total population in Hawai‘i) for gout and RA, using US census population in Hawai‘i for each year [9]. The trends of health care utilization were evaluated using the Cochran–Armitage test for trend to test if the rates of health care utilization have changed over time. We assessed cost data for gout for outpatient visits, inpatient visits, and emergency department visits. The cost data consisted of the primary direct fixed cost and the primary direct variable cost. Primary direct fixed cost consisted of the cost of salaried labor, buildings, and equipment. Primary direct variable cost consisted of the cost of medication and supplies [10]. We calculated mean cost per visit for outpatient visits, emergency department visits and inpatient visits. Difference in the cost between rheumatoid arthritis and gout patients were assessed using Mann Whitney U test. All p-values were 2-sided with a significance threshold of p <0.05. Statistical analyses were performed using R Version 3.4.3 (R Development Core Team, 2017).

## Results

### Characteristics of the study population

The characteristics of patients with gout and rheumatoid arthritis are summarized in **Table 1**.

**Table 1:** Characteristics of gout and rheumatoid arthritis patients 2011–2017.

Gout							
Year	2011	2012	2013	2014	2015	2016	2017
**Discharges, No**	1496	1644	1571	1388	1343	1028	1082
**Age** (years)							
18–44	23.1	21.5	22.0	23.8	24.6	22.7	23.4
45–64	33.1	37.3	33.0	31.3	30.3	30.6	29.0
65–84	38.8	34.2	36.8	37.5	39.4	39.9	41.5
85+	5.0	6.9	8.2	7.3	5.7	6.8	6.1
**Sex** (%)							
Male	84.5	84.5	83.6	82.4	80.9	83.2	86.0
Female	15.5	15.5	16.4	17.6	19.1	16.8	14.0
**Race (%)**							
White	16.0	15.1	16.4	14.6	13.6	14.6	13.7
African-American	0.5	0.7	0.7	0.4	0.6	1.0	1.0
Native Hawaiian and other Pacific Islander	32.5	30.8	30.9	30.2	28.4	31.6	29.9
Asian-American	44.0	48.0	45.8	47.0	51.0	46.6	50.4
Hispanic	1.3	1.0	1.7	2.9	2.8	2.5	2.3
Unknown/Other	5.7	4.4	4.5	5.0	3.5	3.7	2.7
**Payment** (%)							
Medicare	47.4	44.5	48.1	47.2	46.8	45.2	49.2
Medicaid	15.0	15.1	14.3	17.7	14.2	16.6	16.5
Private insurance	35.4	38.7	35.9	33.9	38.0	36.4	33.5
Self-pay/Other	2.1	1.6	1.8	1.3	1.0	1.8	0.9
**Rheumatoid Arthritis**							
**Year**	2011	2012	2013	2014	2015	2016	2017
**Discharges, No**	1184	1261	1124	1007	1038	718	940
**Age** (year)							
18–44	9.6	12.1	11.7	9.1	5.8	2.9	4.4
45–64	31.7	28.5	27.0	29.1	23.4	24.9	23.0
65–84	49.3	50.8	52.8	52.6	58.6	56.0	59.8
85+	9.4	8.6	8.5	9.1	12.2	16.2	12.9
**Sex** (%)							
Male	22.4	21.8	23.6	21.2	24.4	21.0	21.5
Female	77.6	78.2	76.4	78.8	75.6	79.0	78.5
**Race (%)**							
White	23.6	24.6	24.4	23.5	25.0	23.5	24.5
African-American	1.4	0.6	0.3	0.4	0.7	1.1	0.7
Native Hawaiian and other Pacific Islander	12.2	13.1	11.5	13.0	10.1	10.3	9.6
Asian-American	54.2	51.9	54.6	54.7	57.3	58.5	58.1
Hispanic	3.1	3.0	2.5	3.1	2.9	4.2	3.0
Unknown/Other	5.6	6.9	6.8	5.3	4.0	2.4	4.1
**Payment** (%)							
Medicare	61.7	62.6	62.5	64.6	71.1	73.5	73.3
Medicaid	6.9	6.9	4.7	4.4	2.6	3.3	3.4
Private insurance	31.2	30.3	32.7	31.0	26.2	23.1	23.3
Self-pay/Other	0.3	0.2	0.2	0.0	0.1	0.0	0.0

In 2017, approximately 86% of patients with the primary discharge diagnosis of gout were male, and 75% of patients were aged 45 and older. The patients consisted mainly of Asian Americans (50.4%) and Native Hawaiians (29.9%). These proportions were stable with no apparent change during the study period from 2011–2017. Approximately 66% of patients had a primary payer which was either Medicare or Medicaid.

There were a total of 9552 patient encounters (consisting of outpatient visits, inpatient admissions, and emergency department visits) with a primary discharge diagnosis of gout from 2011–2017.

For a primary discharge diagnosis of gout, in 2017, there were 686 outpatient visits, 387 emergency department visits, and nine hospital admissions. As compared to 2011, the number of outpatient visits (p<0.0001), emergency department visits (p<0.0001), and hospital admissions (p = 0.0018) were decreasing.

Seventy-nine percent of RA patients were female, and 95% were 45 years and older in 2017, which was slightly higher than in earlier years (e.g., 90% in 2011). The patients consisted mainly of Asian Americans (58.1%), White (24.5%), followed by Native Hawaiians (9.6%). Approximately 77% of RA patients had Medicare or Medicaid as the primary payer.

There were a total of 7272 patient encounters (consisting of outpatient visits, inpatient admissions, and ER visits) with a primary discharge diagnosis of RA from 2011–2017. For a primary discharge diagnosis of RA, in 2017, there were 926 outpatient visits, ten emergency department visits, and four hospital admissions. As compared to 2011, the number of outpatient visits decreased (p<0.0001) while the number of emergency department visits (p = 0.4749) and hospital admissions (p = 0.1882) was relatively stable.

For patients with a primary discharge diagnosis of gout in 2017, 36% of the total number of encounters consisted of emergency department visits, while only 1% of the total number of encounters consisted of emergency department visits for patients with a primary discharge diagnosis of RA.

### Annual rates of outpatient visits, inpatient admission and emergency department visit of gout and RA

For patients with a primary discharge diagnosis of gout, the annual rates for emergency department visits, outpatient visits, and inpatient admissions throughout the 7-year period were trending downward and significant from 2011 to 2017 (p <0.05) **(Table 2)**. Emergency department visits decreased from 0.33 to 0.27 per 1000 Hawai‘i adults (p <0.001) and outpatient visits decreased from 0.73 to 0.48 per 1000 Hawai‘i adults (p <0.001).

**Table 2:** Healthcare utilization of gout and rheumatoid arthritis patients: rates of outpatient visits, admissions, and emergency department visits.

Gout– Patients per 1,000 in Hawai‘i								
**Year**	2011	2012	2013	2014	2015	2016	2017	P-value
**Patients Category**								
Total	1.085	1.180	1.116	0.979	0.942	0.720	0.758	<0.0001
Emergency	0.332	0.411	0.373	0.358	0.299	0.281	0.271	<0.0001
Outpatient	0.737	0.751	0.732	0.610	0.637	0.428	0.481	<0.0001
Inpatient	0.016	0.018	0.011	0.011	0.006	0.011	0.006	0.0018
**Rheumatoid** **Arthritis–** **Patients per 1,000** **in Hawai‘i**								
**Year**	2011	2012	2013	2014	2015	2016	2017	P-value
**Patients Category**								
Total	0.859	0.905	0.798	0.710	0.728	0.503	0.659	<0.0001
Emergency	0.007	0.008	0.008	0.008	0.004	0.005	0.007	0.4749
Outpatient	0.850	0.893	0.788	0.699	0.721	0.497	0.649	<0.0001
Inpatient	0.003	0.005	0.003	0.003	0.002	0.001	0.003	0.1882

**Fig 1:**
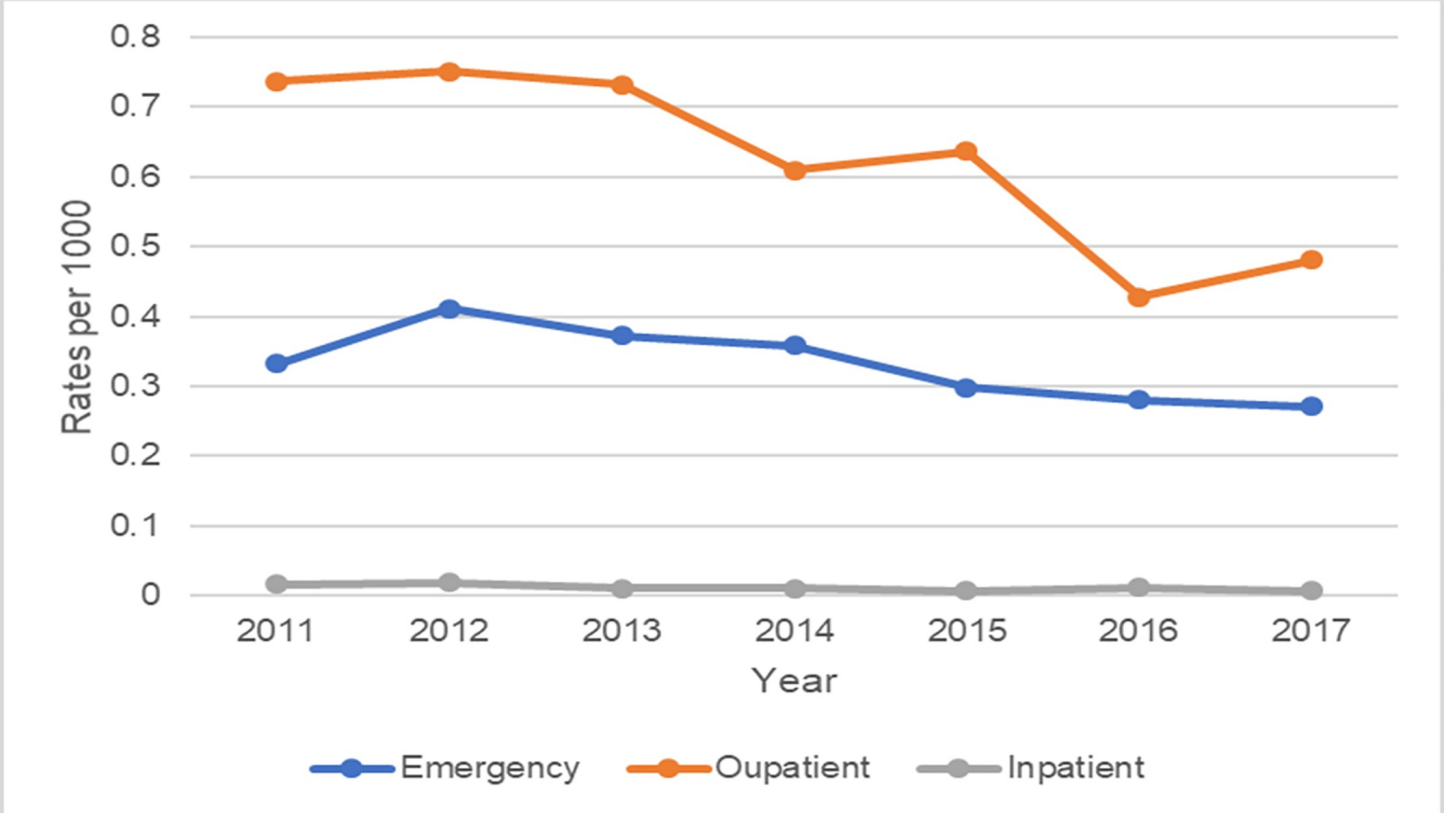
Annual rates of emergency department visits, outpatient visits and inpatient hospitalizations with a primary discharge diagnosis of gout 2011–2017.

**Fig 2:**
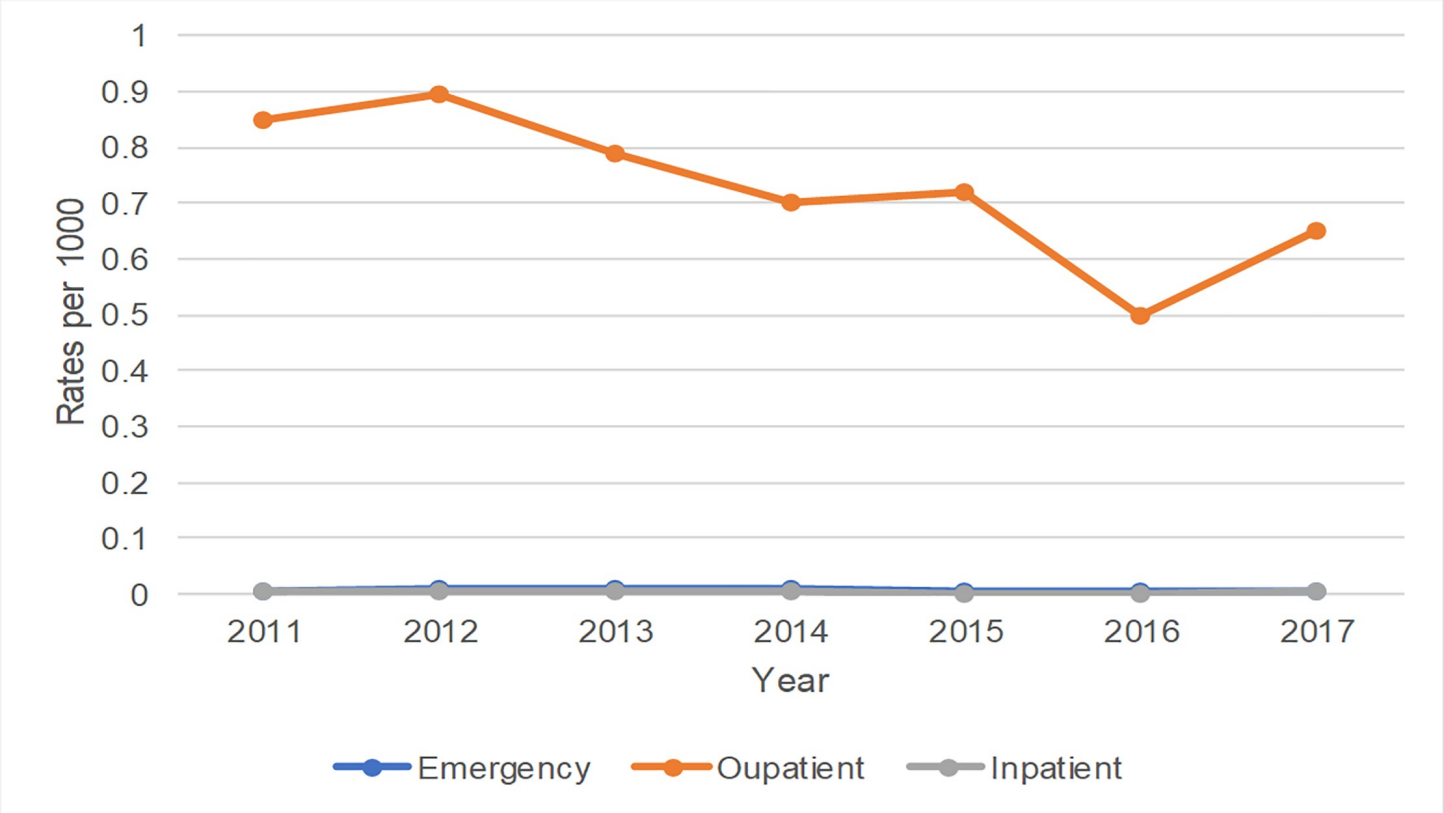
Annual rates of emergency department visits, outpatient visits and inpatient hospitalizations with a primary discharge diagnosis of rheumatoid arthritis 2011–2017.

For patients with a primary discharge diagnosis of RA from 2011 to 2017, the annual rates for emergency department visits, and inpatient admissions were stable and unchanged, while outpatient visits decreased over the 7-years from 2011 (p<0.05). In contrast with gout patients, RA patients had low rates of emergency department visits from 2011–2017, with most of the healthcare utilization focused in the outpatient setting (see Table 2, Fig 1 and Fig 2).

### Costs outpatient visits, inpatient visits, and emergency department visits of gout

In 2017, the mean cost per visit for outpatient visits for gout patients was $54.8. The mean cost per visit for emergency department visits was $183.5, approximately 3.4 times the cost of outpatient visits. The cost per visit for inpatient visits was $6062.9. In 2017, the mean cost per visit for outpatient visits for rheumatoid arthritis patients was $53.4. The mean cost per visit for emergency department visits was $189.2, approximately 3.5 times the cost of outpatient visits. The cost per visit for inpatient visits was $8497.4. The aveRAge cost of outpatient visits, emergency department visits, and inpatient visits did not significantly differ by gout or rheumatoid arthritis patients.

In 2017, the median cost per visit for outpatient visits for gout patients was $23.78 (Interquartile range (IQR) = 36.04). The median cost per visit for emergency department visits was $154.76 (IQR = 74.16), approximately 6.5 times the cost of outpatient visits. The median cost per visit for inpatient visits was $6431.45 (IQR = 4479.87). In 2017, the mean cost per visit for outpatient visits for rheumatoid arthritis patients was $23.78 (IQR = 34.5). The median cost per visit for emergency department visits was $145.45 (IQR = 48.61), approximately 6.1 times the cost of outpatient visits. The median cost per visit for inpatient visits was $8798.10 (IQR = 6377.64). The costs of outpatient visits, emergency department visits, and inpatient visits did not differ by gout or rheumatoid arthritis patients. (p>0.05 for all outpatients, emergency department, and inpatient visits).

## Discussion

Our findings show high rates of emergency department utilization for gout patients within a single healthcare system in Hawai‘i. Although emergency department utilization rates decreased from 2011–2017, emergency department visits represented a significant portion of health care utilization for gout patients. In comparison, RA patients were primarily managed in the outpatient setting, with stable and very low rates of emergency department use. Emergency department visits are associated with higher cost compared to outpatient visits [11]. Our study highlights a gap in the long-term treatment of gout. Efforts are needed to facilitate the outpatient evaluation of gout patients to reduce emergency department utilization to reduce the health care costs of gout patients.

Several studies using United States national databases have shown gout emergency department visits in the United States are associated with significant cost and burden to the health care system [3, 12]. From 2006–2012, the rates of gout emergency department visits in the United States increased from 75.0 to 85.4 per 100,000 persons [3]. Emergency department costs increased from 80% from 156 million to 281 million [3]. Our study from a more recent study period (2011–2017) showed decreasing emergency department visits. Despite this decrease, ER visits constituted a significant portion of health care utilization among gout patients, in contrast to RA patients where emergency utilization was low from 2011–2017. The decrease in rates of emergency department visits and inpatient hospitalizations may reflect effects of capitated care being implemented by the biggest insurance provider in the state of Hawai‘i, leading to efforts in reducing costly emergency department visits and in hospital admissions [13].

Many emergency department visits are preventable, and they may indicate suboptimal care, inadequate access to care or, poor choices on the part of patients [14]. Prior studies have documented several gaps in the chronic management of gout care. For example, gout patients who fulfill the indication for urate-lowering treatment had low rates of initiation of urate-lowering agents [15]. Furthermore, gout patients who were on urate-lowering agents commonly had inadequate serum urate testing and inadequate dosing of urate-lowering agents [16]. This gap may be due to a poor understanding of therapeutic objectives in reducing serum uric acid levels among health care providers [17]. Other important gaps include poor patient adherence to treatment [18] and the patient’s lack of knowledge and education in regards to their diagnosis and management of disease [19]. Efforts are required to bridge these gaps in care including improving the education of providers and patients, engaging primary care physicians in adopting effective ambulatory models of urate lower treatment initiation, and broadening the use of allied health professional-run gout clinics [20].

Improving access to care for gout patients, especially during a gout flare is an essential step in reducing emergency department visits. Efforts to improve access in primary care include training to increase the primary care physician’s knowledge to manage gout flares, extension of primary care operating hours, and implementation of managed care on emergency department use (capitated payment of the primary care physician, or requiring primary care physician approval for emergency department use) [21]. Providing increased acute care outside the emergency department via urgent care centers and outpatient retail clinics may be an effective strategy in reducing costly emergency department visits [21]. Urgent care centers and outpatient retail clinics can manage an estimated 13–27% of emergency visits in the United States, with significant potential cost savings [21].

Rheumatoid arthritis was primarily managed in the outpatient setting, with low rates of emergency department and inpatient admissions. This pattern of health care utilization is consistent with prior studies showing a decline in inpatient admissions for RA which were previously attributed to complications of RA (Felty’s syndrome, rheumatoid vasculitis) and joint replacements [22–24]. Improvement in RA care in recent years may have contributed to these positive findings. These improvements include efforts and medical advancements that facilitate prompt and accurate diagnosis and treatment [25], the introduction of effective treatments (methotrexate, biologics) [26, 27], and advancement in new treatment paradigms (‘treat to target’ strategies aiming at remission) [28, 29].

Studies of gout in Hawai‘i were mainly performed in the 1950s-1960s with no subsequent follow-up studies [30–32]. Our study provides recent and contemporary information about gout care in Hawai‘i, information which has been significantly lacking despite its importance given the many ethnocultural groups in Hawai‘i including Native Hawaiians and Asian Americans who are thought to have increased the risk of uric acid levels and gout [6]. Notably, Asian American and Native Hawaiian patients consisted of approximately 50% and 30% of gout patients respectively in our study, while only comprising only of 38.6% and 10% of the total population in Hawai‘i [9]. This suggests a higher prevalence of gout, and possibly a disparity of quality of care in these populations meriting further investigation. With increasing gout prevalence and the association of gout with the significant economic burden and the decreased quality of life for the Hawai‘i population, we need further studies to inform evidence-based health policies and practices in Hawai‘i [6].

There are several limitations to our study. Our study is limited to a single large health care system in Hawai‘i. Therefore our findings may not be generalizable to other health care systems [33]. Nevertheless, it does complement prior studies about suboptimal gout care, and as previously mentioned provides important information about gout care in Hawai‘i. Secondly, there is a possibility of misclassification of diagnostic codes used to identify gout and RA patients. To minimize this, we included only patients with a primary discharge diagnosis of gout or RA. We excluded patients who had less than a one-year follow-up from the study. Prior studies have used similar methodologies, to study the emergency department and inpatient gout admissions [1, 3, 12].

In summary, our study highlights a significant gap in gout care in Hawai‘i and high rates of emergency department visits of gout patients, in comparison to RA. Because emergency department visits are costly, further steps are needed to improve the quality of chronic gout management. Increased access to care for gout patients via primary care, and non-emergency department acute clinics, and improved patient education is needed to improve care to reduce the cost to the health care system.
